# Association between serum folate and T cell subsets in a population-based study

**DOI:** 10.1007/s00394-025-03872-x

**Published:** 2026-01-16

**Authors:** Laura Vanessa Stoß, Christa Meisinger, Florian Rohm, Jakob Linseisen, Dennis Freuer

**Affiliations:** 1 Epidemiology, Medical Faculty, University of Augsburg, Augsburg, Germany; 2 Institute for Medical Information Processing, Biometry and Epidemiology (IBE), Faculty of Medicine, LMU, Munich, Germany; 3 Pettenkofer School of Public Health, Munich, Germany

**Keywords:** MEIA, Folate, Vitamin B9, T cell subsets, Adaptive immunity, Nutritional immunity

## Abstract

**Purpose:**

Folate, also called vitamin B9, plays a key role in various cellular processes that support T cell proliferation and function, which in turn regulate adaptive immune responses. Nonetheless, the associations between serum folate concentrations and specific T cell subsets remain underexplored.

**Methods:**

Data of the population-based MEIA study were analyzed including 594 participants aged 19–75 years in Augsburg study region, Germany. Serum folate concentrations were measured (right-censored at 20 ng/mL) and immunophenotyping of T cells via flow cytometry was performed in fresh blood samples. Associations between folate concentrations and T cell subsets were analyzed using a multivariable two-stage regression model to account for censoring.

**Results:**

Positive associations were noted with Effector Memory CD8 + T cells ($\:\beta\:$= 0.39; 95% CI [0.09;0.70]), CD8+ CD27- CD28+ T cells ($\:\beta\:$= 1.02; 95% CI [1.00; 1.03]), and CD8+ CD27+ CD28- T cells ($\:\beta\:$= 1.01; 95% CI [1.00; 1.03]), negative associations were observed with Central Memory CD8+ T cells ($\:\beta\:$= − 0.29; 95% CI [− 0.47; − 0.10]) and naïve CD8+ T cells ($\:\beta\:$= − 0.33; 95% CI [− 0.63; − 0.04]). Although these associations did not retain statistical significance after adjustment for multiple testing, they were fully supported by sensitivity analyses.

**Conclusion:**

This study provides preliminary evidence linking serum folate to specific T cell subsets, particularly within CD8 + populations. While findings are suggestive, they emphasize the potential importance of adequate folate levels for immune health. Further longitudinal and interventional studies are needed to confirm these associations and explore the role of folate in immune function.

**Supplementary Information:**

The online version contains supplementary material available at 10.1007/s00394-025-03872-x.

## Introduction

Folate, also known as vitamin B9, is a water-soluble vitamin essential for various cellular functions. Folate occurs naturally in foods such as beans and dark green leafy vegetables. In a synthetic form, folic acid is used in supplements and fortified foods due to its higher bioavailability and stability [1–5]. Folate deficiency is commonly defined as a serum folate concentration below 3 ng/mL (6.7 nmol/L) or a red blood cell (RBC) folate concentration below 134 ng/mL (305 nmol/L) [6–8]. The recommended dietary allowance (RDA) for adults is 400 µg dietary folate equivalents (DFE) per day [9]. Global estimates indicate that folate deficiency affects approximately 20–25% of women of reproductive age in low- and middle-income countries (LMICs) and about 5–10% in high-income countries [10]. In Germany, the folate levels of approximately 14% of the adult population are inadequate. Populations most at risk for inadequate levels are women of reproductive age and individuals with lower socioeconomic status [8]. Folate deficiency has been associated with neural tube defects, Alzheimer’s disease, and certain types of cancers [11–13], highlighting its importance in human health. Folate has an important role in nucleotide biosynthesis, DNA repair, and methylation - processes essential for genomic stability [14, 15]. It also plays a role in mitochondrial function and maintenance of mitochondrial DNA [16]. Furthermore, it was shown that folate-deficient subjects had approximately two times higher plasma homocysteine levels compared to controls. Elevated plasma homocysteine concentrations are associated with a higher risk of cardiovascular disease, peripheral arterial disease, stroke, and venous thrombosis [17].

These processes are vital for the proliferation and function of T cells, a subset of lymphocytes in the adaptive immune system, crucial for antigen-specific immunity and immunological memory. T cells originate in the bone marrow, mature in the thymus gland [18], and are classified in different types: Cytotoxic CD8 + T cells target virus-infected and cancerous cells, while CD4 + helper T cells support B and CD8 + T cells [19]. Regulatory T cells (Tregs) are crucial for maintaining immune tolerance and preventing autoimmune responses. Recent studies have identified folate receptor 4 as a marker strongly expressed on Tregs, which suggests a link between folate metabolism and Treg function [20, 21]. This connection and other emerging evidence suggest that low folate may have an impact on the adaptive immune system, particularly T cell function [21–24]. However, the specific associations remain to be understood. By investigating the relationships between serum folate levels and T cell populations in peripheral blood, this population-based study aims to provide insights into the role of folate for immune modulation; possibly, it may also form a basis for future studies to investigate the potential for targeted nutritional interventions to enhance immune health.

## Methods

### Study design and population

The data used in these analyses originated from the population-based “Metabolism, nutrition, and the immune system in Augsburg” (MEIA) study. The MEIA study included 594 participants randomly drawn from the population registries. Participants were 19 to 75 years old and lived (with the main residence) in the city of Augsburg or the two adjacent counties Aichach-Friedberg and Augsburg, which altogether form the Augsburg study region. All individuals who were unable to provide informed consent were excluded. This could be due to a lack of knowledge of the German language or due to handicaps that precluded full understanding of the study information. We did not exclude women or men according to their supplement intake.

### Data collection

In the MEIA study, data and biospecimens, including blood, urine, and stool samples, were collected between April 22, 2021, and July 14, 2023. Participants filled in standardized questionnaires and were interviewed regarding, e.g., socio-demographics, lifestyle habits, and their medical history such as previous diabetes diagnosis. Additionally, participants were invited to undergo physical and cognitive assessments, including anthropometric measurements and bioelectrical impedance analysis (BIA). All examinations were conducted by trained and certified study personnel. Data collection adhered to the standards set by the Declaration of Helsinki, with participants providing written informed consent. They were informed about the study’s procedures and potential consequences. Approval for the study was granted by the Ethics Committee of Ludwig-Maximilian-Universität Munich.

### Laboratory measurements

Blood samples were collected in an overnight fasting state (at least 10 h) and handled in accordance with a standardized operating procedure (SOP). Standard laboratory parameters, like blood lipids concentrations and serum folate levels were measured after SOP-compliant preanalytical treatment immediately in the laboratory of University Hospital of Augsburg. Serum folate levels were not determined in hemolytic blood samples. The electrochemiluminescence immunoassay “ECLIA” was used to measure serum folate. The measurement was performed on the analyzer cobas e801 from ROCHE. Serum folate values were right censored by the laboratory at 20 ng/mL, with all values above that threshold being set to 20 ng/mL. Additionally, immunophenotyping of participants was performed using venous EDTA whole blood samples. This involved the differentiation and quantification of various leukocyte subpopulations through flow cytometry with fluorescence-labeled antibodies, also known as FACS (fluorescence-activated cell sorting). The analysis was conducted using samples prepared with the DURAClone IM T Cell Subsets and DURAClone IM Treg kits from Beckman Coulter, using the Navios Flow Cytometer. Data analysis followed the manufacturer’s instructions and was performed using the Kaluza software, provided by Beckman Coulter. A detailed description of the flow cytometry and the applied gating strategy can be found in the supplementary information (*Supplementary Method*).

### Statistical analysis

Baseline characteristics were presented as absolute frequencies and percentages for categorical variables and as means with standard deviations as well as medians along with their corresponding interquartile ranges for continuous variables. Differences between male and female participants were assessed using the Chi-square test for categorical data or the two-sided Mann-Whitney U test and two-sided t-test for non-normally and normally distributed continuous data, respectively. The distribution was examined visually with histograms and QQ-plots, as well as the Kolmogorov-Smirnov test. Outliers in the outcome variables, defined as values differing from the mean by more than three standard deviations, were excluded.

To investigate the association between folate (exposure) and T cells (outcomes) a two-stage multivariable linear regression model was used due to the right-censoring of the serum folate data. We opted for this two-stage regression approach instead of imputing values above the threshold for the right-censored data, as extrapolation can lead to seriously biased estimates, potentially distorting the associations under investigation [25]. The first stage of the regression considered folate values below 20 ng/mL. In the second stage, the regression was performed using a binary exposure reflecting whether the threshold was reached. Regarding the first stage, the associations between serum folate concentrations and the relative frequencies of gated T cell subsets were initially modelled using multivariable linear regression models. Depending on the distribution of the residuals, an outcome was log-transformed if this improved the fit regarding a normal distribution. If the assumption of normally distributed residuals could not be met in either approach, median regression was employed instead. Homoscedasticity and the normal distribution of residuals in the parametric models were evaluated visually through residual scatter plots (comparing standardized residuals to fitted values) and Q-Q plots, respectively. Outliers exceeding a Cook’s Distance of 0.5 were excluded as well if they seemed to distort the model.

Confounders were determined using directed acyclic graphs based on evidence from the literature [26–29]. Consequently, data on age, sex, education (International Standard Classification of Education ISCED3C), smoking intensity (pack years: number of packs smoked per day multiplied with the number of years), alcohol consumption (AUDIT score), relative body fat content, and Low-Density Lipoprotein (LDL) cholesterol levels were included in the models to account for potential confounding factors. The AUDIT-C score is a validated screening instrument that evaluates drinking patterns on a scale from 0 to 12, with higher scores reflecting increased alcohol intake and associated risk. As only two participants supplemented folic acid, this information was not included as a confounder. To test the linearity assumption, restricted cubic splines for the continuous independent variables were used and the optimal number of knots between three and five was determined for each continuous independent variable based on the Akaike Information Criterion and were presented in Supplementary Table 1. Missing covariate data was assumed to follow a random or completely random pattern. To address this, multivariate imputation by chained equations with five imputations was applied.

Using a type I error probability of 0.05, the reported p-values were adjusted for multiple testing by applying the false discovery rate (FDR) method. P-values significant before but not after adjusting for multiple testing will be interpreted in the following as suggestively significant. The derived effect estimates ($\:\beta\:$, 95% Confidence Interval) from parametric models can be interpreted as the expected change in the outcome variable associated with one unit increase in serum folate concentration. Median regression estimates represent analogously the median of changes of the outcome variable. Effect modification was assessed by testing the interaction between the primary exposure (folate) and the selected confounders age, sex, and physical activity.

Additionally, to address the potential impact of outlier exclusion, we conducted a sensitivity analysis. This involved excluding only extreme values categorized as outliers, rather than those differing from the mean by more than three standard deviations, and re-running the regression analysis on this adjusted dataset.

The statistical analyses were conducted using the statistical software R version 4.4.1 using mainly the packages dplyr (1.1.4), ggplot2 (3.5.1), tidyr (1.3.1), stringr (1.5.1), tableone (0.13.2), knitr (1.47), memisc (0.99.31.7), psych (2.4.6.26), visdat (0.6.0), DataExplorer (0.8.3), Hmisc (5.1-3), openxlsx (4.2.5.2), kableExtra (1.4.0), quantreg (5.98), writexl (1.5.0), mice (3.16.0), MASS (7.3–60.2), lmtest (0.9–40), car (3.1-2), ggfortify (0.4.17), and rms (6.8-2).

## Results

### Baseline characteristics of the study population

Only 14 participants had serum folate concentrations below 3.0 ng/mL indicating folate deficiency. A right-censored threshold of 20 ng/mL was observed in 44 participants.

Continuous characteristics of the MEIA study sample stratified by sex are presented in Table 1. A total of 594 participants with a mean age of 47.37 years were included in the study, comprising 258 men (mean age: 47.74 years) and 336 women (mean age: 47.09 years). The median serum folate concentration differed between male and female participants with male median levels being at 7.40 ng/mL (IQR: 5.40–10.12) and female median levels at 9.40 ng/mL (IQR: 6.60–13.40). LDL cholesterol levels did not differ significantly between sexes, whereas High-Density Lipoprotein (HDL) cholesterol levels were higher in women. Body Mass Index (BMI) was higher in men, while women had a greater relative fat mass. With a median of 4.00 the AUDIT-C Score was higher in men than in women, where a median of 2.00 was observed. Categorical characteristics of the study population stratified by sex are presented in Table 2. No differences between the sexes were found regarding smoking status, physical activity and education level.

**Table 1:** Continuous characteristics of the “Metabolism, nutrition and immune system in Augsburg” (MEIA) study population stratified by sex

Characteristics	Total (*n* = 594)		Men (*n* = 258)		Women (*n* = 336)		*p*-value
	Mean (SD)	Median (IQR)	Mean (SD)	Median (IQR)	Mean (SD)	Median (IQR)	
Age (years)	47.37 (14.88)	49.00 (34.00; 60.00)	47.74 (15.24)	50.00 (34.00; 61.00)	47.09 (14.61)	49.00 (34.00; 58.00)	0.485^a^
BMI (kg/m^2^)	26.38 (5.19)	25.59 (22.64; 29.00)	27.34 (4.48)	26.75 (24.55; 29.45)	25.64 (5.56)	24.34 (21.24; 28.30)	< 0.001^b^
Waist circum- ference (cm)	88.25 (15.27)	87.00 (76.00; 100.00)	96.10 (13.68)	96.00 (86.00; 103.75)	82.23 (13.62)	79.00 (71.00; 91.00)	< 0.001^b^
LDL cholesterol (mg/dL)	120.04 (34.01)	118.00 (96.00; 142.00)	120.93 (33.67)	121.00 (99.00; 142.75)	119.35 (34.31)	116.50 (92.00; 142.00)	0.575^a^
HDL cholesterol (mg/dL)	62.27 (16.99)	60.50 (50.00; 72.00)	54.27 (13.58)	52.00 (45.00; 63.00)	68.49 (16.79)	66.00 (57.00; 78.00)	< 0.001^a^
Relative Fat Mass (% of Body Weight)	31.02 (8.90)	30.21 (24.69; 36.81)	26.18 (7.17)	26.22 (21.56; 30.85)	34.82 (8.27)	34.35 (28.48; 40.97)	< 0.001^a^
Alcohol Total Score	3.06 (2.18)	3.00 (1.00; 4.00)	3.75 (2.36)	4.00 (2.00; 5.00)	2.53 (1.86)	2.00 (1.00; 4.00)	< 0.001^b^
Serum folate (ng/mL)	9.47 (4.77)	8.25 (6.00; 11.60)	8.20 (3.98)	7.40 (5.40; 10.12)	10.45 (5.09)	9.40 (6.60; 13.40)	< 0.001^b^

Alcohol Total Score was measured using the AUDIT-C screening tool, which assesses alcohol consumption through a score ranging from 0–12. Higher scores indicate higher levels of alcohol consumption. The mean (Standard Deviation, SD) and median (Interquartile Range, Q1; Q3) were presented. * P*-values were determined using the t-test^a^ for normally distributed variables and the Mann-Whitney U test^b^ for those not normally distributed. BMI: body mass index, HDL: High-Density Lipoprotein, LDL: Low-Density Lipoprotein

**Table 2:** Categorical characteristics of the “Metabolism, nutrition and immune system in Augsburg” (MEIA) study population stratified by sex

Characteristics	Total (n = 594)	Men (n = 258)	Women (n = 336)	*p*-value
Smoking				
Current	96 (16.16%)	46 (17.83%)	50 (14.88%)	0.227
Never	300 (50.51%)	120 (46.51%)	180 (53.57%)	
Previous	198 (33.33%)	92 (35.66%)	106 (31.55%)	
Physical activity level				
Sedentary	109 (18.83%)	39 (15.66%)	70 (21.21%)	0.088
Low active	177 (30.57%)	70 (28.11%)	107 (32.42%)	
Active	164 (28.32%)	75 (30.12%)	89 (26.97%)	
Very active	129 (22.28%)	65 (26.10%)	64 (19.39%)	
Diabetes status				
No diabetes	564 (96.25%)	242 (95.65%)	322 (96.70%)	0.660
Has diabetes	22 (3.65%)	11 (4.35%)	11 (3.30%)	
Education				
Lower education	233 (39.23%)	110 (42.64%)	123 (36.61%)	0.199
Middle education	333 (56.06%)	134 (51.94%)	199 (59.23%)	
Higher education	28 (4.71%)	14 (5.43%)	14 (4.17%)	

Variables were displayed as n (column %) and analyzed using the Chi-Square Test

### Main analyses

**Fig. 1:**
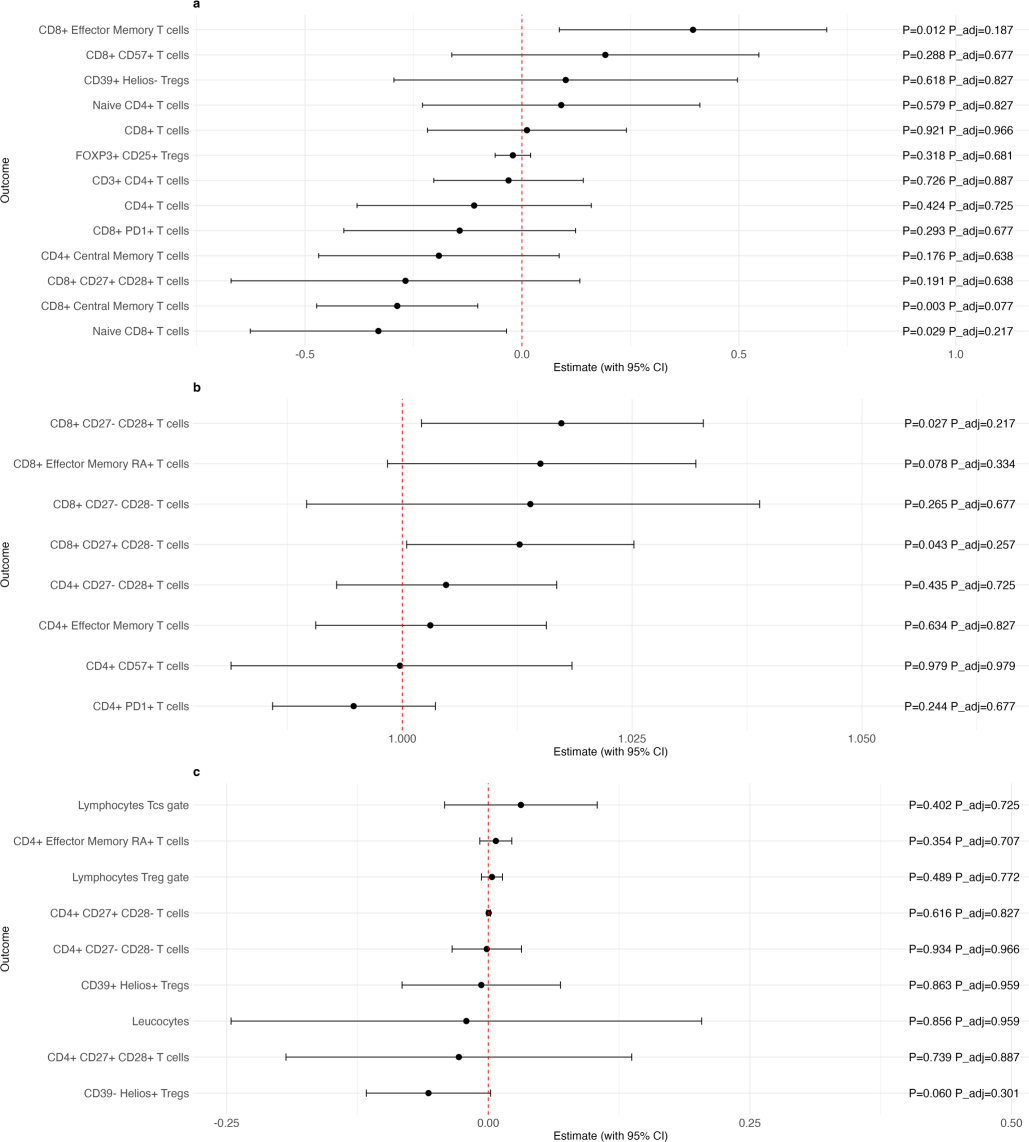
Association between serum folate concentrations (Sub-20 B9) and the relative frequencies of gated T cell subsets, expressed as percentages of cells within the corresponding gate. Estimates and 95% confidence intervals were calculated from the linear regression models (Panel a), the log-linear regression models (Panel b) and median regression models (Panel c), adjusting for confounding variables: age, sex, smoking status, alcohol consumption, education level, LDL cholesterol, and relative body fat percentage. * P*-values are presented both before (“P”) and after correction for multiple testing using the FDR method (“P_adj.”). * Abbreviations*: CI = confidence interval, Tregs = Regulatory T cells

**Fig. 2:**
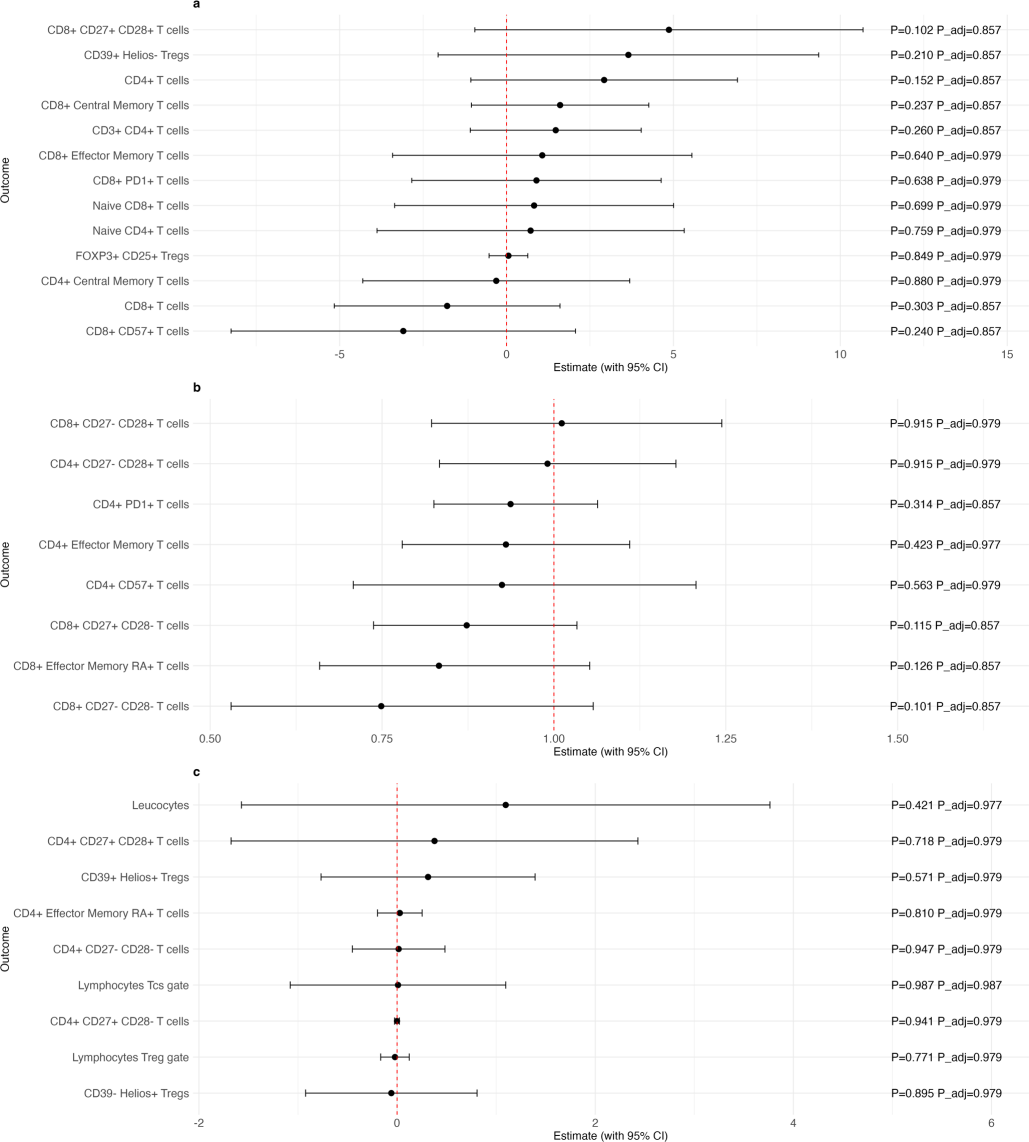
Association between serum folate concentrations (Threshold B9) and the relative frequencies of gated T cell subsets, expressed as percentages of cells within the corresponding gate. Estimates and 95% confidence intervals were calculated from the linear regression models (Panel a), the log-linear regression models (Panel b) and median regression models (Panel c), adjusting for confounding variables: age, sex, smoking status, alcohol consumption, education level, LDL cholesterol, and relative body fat percentage. P-values are presented both before (“P”) and after correction for multiple testing using the FDR method (“P_adj.”). Abbreviations: CI = confidence interval, Tregs = Regulatory T cells

Figures 1 and 2 present the associations between serum folate concentrations and various T cell subtypes in peripheral blood, adjusted for potential confounders, including age, sex, education, smoking, alcohol consumption, relative body fat distribution and LDL cholesterol levels. The forest plots display effect estimates with their 95% confidence intervals, alongside both unadjusted (“p”) and adjusted p-values (“p adj.“) from the regression analyses. Figure 1 presents the results from the first stage regression model using the continuous folate variable, limited to values below 20 ng/mL (Sub-20 B9), while Fig. 2 illustrates estimates from the second stage model using the binary threshold for folate (Threshold B9). In both figures, Panel A displays the effect estimates derived from linear models, Panel B from log-linear models, and Panel C from median regression analyses.

Figure 1 highlights suggestive positive associations for Sub-20 B9 with the relative frequencies of Effector Memory (EM) CD8 + T cells ($\:\beta\:$ = 0.39; 95% CI [0.09; 0.70]; *p* = 0.012; p adj. = 0.187), log-transformed CD8+ CD27- CD28+ T cells ($\:\beta\:$ = 1.02; 95% CI [1.00; 1.03]; *p* = 0.027; p adj. = 0.217) and log-transformed CD8+ CD27+ CD28- T cells ($\:\beta\:$ = 1.01; 95% CI [1.00; 1.03]; *p* = 0.043; p adj. = 0.257). A negative suggestive association was found with Central Memory (CM) CD8+ T cells ($\:\beta\:$ = − 0.29; 95% CI [− 0.47; − 0.10]; *p* = 0.003; p adj. = 0.077) and naïve CD8+ T cells ($\:\beta\:$ = − 0.33; 95% CI [− 0.63; − 0.04]; *p* = 0.029; p adj. = 0.217). The median regression revealed no associations of folate and the respective outcomes.

No notable associations between folate levels and T cell subtypes could be observed from the second-stage regression using the Threshold B9 as exposure (Fig. 2). Comparing the effect estimates for suggestive associations across the two stages reveals consistent directions for some T cell subtypes, yet inconsistencies for others. Specifically, the estimates for CM CD8 + T cells were negative in both the Sub-20 B9 and Threshold B9 analyses, while for CD8 + CD27- CD28 + T cells, both estimates were positive across the two stages. However, for the remaining suggestive associations, EM CD8 + T cells, naïve CD8 + T cells, and CD8 + CD27 + CD28- T cells, the direction of effect estimates differed between the two stages. All effect estimates are provided in Supplementary Tables 2 and 3.

### Sensitivity analyses

The tested interaction terms, namely between the primary exposure variables (Threshold B9 and Sub-20 B9) and key confounders (such as age, sex, and physical activity), which were included to test for potential moderator effects, did not significantly improve the models. The absence of notable interaction effects indicated that the associations between serum folate and T cell outcomes were not differential between confounder expressions. The sensitivity analysis, conducted by excluding only extreme outliers, yielded results consistent with those of the main analysis. Detailed results are presented in Supplementary Tables 4 and 5. This approach addressed outliers with a strong influence on the models while keeping data points that might reflect natural variation in the population. Re-running the regression analysis on the adjusted dataset confirmed that the associations observed in the main analysis were not heavily affected by stricter outlier exclusion criteria. This consistency supports the reliability of the main effect estimates and adds confidence to the interpretation of our results.

## Discussion

This study found suggestive associations between serum folate concentrations and the relative frequencies of various T cell subsets in peripheral blood. More precisely, there were suggestive positive associations between folate levels and specific T cell subsets, such as EM CD8 + T cells, CD8 + CD27- CD28 + T cells and CD8 + CD27 + CD28- T cells. Suggestive negative associations were observed with CM CD8 + T cells and naïve CD8 + T cells. These findings suggest that folate levels may affect certain T cell populations more than others. The lack of significant results following the FDR adjustment indicates the need for cautious interpretation and highlights the importance of further studies to strengthen this preliminary evidence.

Previous studies that investigated associations between certain T cell populations and folate similarly found that folate deficiency inhibits CD8 + T cell and Treg activity as well as proliferation [21, 22]. This has been shown in studies with mice [21, 30] and in vitro with human cells [22]. The consistent estimates from our two-stage approach (i.e. Sub-20 B9 and Threshold B9) regarding CD8 + CD27- CD28 + T cells are in accordance with these findings on CD8 + T cells [21, 22], while the other suggestive associations are not. Two studies focused on broader populations, such as Tregs, CD8+, and CD4 + T cells, examining their subsets in less detail compared to our approach [21, 22]. Building on these broader findings, one study concentrating on T cell subsets similar to our study, found that folate-deficient mice exhibited a significant increase in naïve CD4 + T cells and a decrease in memory and effector CD4 + T cell populations, highlighting the impact of folate deficiency on T cell differentiation [30]. In contrast to past findings, our study did not produce conclusive results for these T cell subsets. Another study in mice found that folic acid supplementation, and thus higher levels of folate, supports FoxP3 + Treg proliferation in the colon [23]. In contrast to these studies highlighting the beneficial effects of folate, an in vivo study in humans from 2022 found that elevated folate levels by high folic acid intake can cause DNA damage in lymphocytes, leading to genomic instability [24]. This study was conducted with a relatively small sample size of only 33 participants, which limits the conclusiveness of its findings. However, the observation of higher folate levels through folic acid intake potentially having negative impacts on lymphocyte populations aligns partially with our results, particularly the consistent negative suggestive associations observed with CM CD8 + T cells. Countries like the US and Canada have mandatory folate fortification and the overall evidence supports this as a successful public health intervention [31]. However, there is no mandatory folic acid fortification of food in Germany. While in our study only two participants reported supplementing folic acid, the synthetic folate is voluntary added to many foods in Germany such as cereals, beverages and dairy products [32], which could explain our results. Folate plays a critical role in DNA synthesis, repair, and methylation [33]. These processes are essential for the proliferation and function of rapidly dividing cells such as T cells. Folate deficiency has been shown to induce cell cycle arrest and apoptosis in T cells, potentially altering the distributions of T cell subsets [22, 34]. Courtemanche et al. investigated the effect of folate deficiency on the in vitro proliferation of primary human CD8 + T lymphocytes [22]. They found that folate deficiency selectively inhibits the proliferation of CD8 + T cells. This impairment was accompanied by S phase cell cycle arrest, increased apoptosis, and marked uracil accumulation in nuclear DNA of CD8 + cells. In addition, folate deficiency increased the ratio of CD4 + to CD8 + cells due to a significant reduction in CD8 + cell proliferation However, when folate was added to folate-deficient T lymphocytes, proliferation and normal cell cycle were rapidly restored. Overall, these results suggest that folate deficiency may affect the immune system by impairing CD8 + T cell function through several molecular mechanisms, primarily by disrupting cell proliferation and DNA synthesis. These findings align with our observations of suggestive associations between folate levels and specific CD8 + T cell subsets, as folate-dependent processes are essential for maintaining T cell homeostasis [33]. Further investigations in larger studies are needed to confirm and clarify the associations identified. The strengths of this study lie in a relatively large sample size of 594 participants from the general population enhancing its statistical power. This sample size is notably larger than many existing studies, focusing on micronutrient and immune associations [24]. To our knowledge, our study is the first to analyze thirty specific T cell subtypes, offering a detailed insight into the immunomodulatory effects of folate beyond general T cell populations. Furthermore, we adjusted for multiple potential confounders. The robustness of our findings was supported by sensitivity analyses, which demonstrated consistent results even when extreme outliers were excluded. This strengthens the validity of the observed associations between serum folate and T cell subsets. The applied statistical methods enhanced the precision of results by minimizing bias.

However, this study presents a set of limitations. The cross-sectional approach of the study design hinders our ability to make causal inferences. While suggestive associations between folate levels and T cell subsets were observed, it is unclear whether variations in serum folate directly influence T cell frequencies or if the frequencies are influenced by other underlying biological mechanisms. Measurement bias due to right-censoring of folate data remains a limitation, as it restricted our ability to analyze the full range of folate levels, potentially obscuring associations with T cell subsets in individuals with high serum folate. Although adjustments were made for several potential confounders, there may still be unmeasured factors, such as genetic variations in folate metabolism (e.g., MTHFR mutations or polymorphisms). RBC folate, which reflects long-term folate intake and body stores, would have provided a more robust measure than serum folate. However, RBC folate was not included in the laboratory parameter portfolio used in the MEIA study. The same states for the homocysteine levels. Additionally, the results are not generalizable to other populations, as the study sample may not reflect the diversity of genetic backgrounds, dietary habits, or environmental exposures present in broader or more heterogeneous groups.

## Conclusion

In summary, this study suggests a potential association between serum folate concentrations and specific T cell subsets, particularly within CD8 + populations, highlighting the importance of adequate folate levels for immune health. While the findings are suggestive rather than definitive, they emphasize the need for further research. Longitudinal studies and intervention trials focusing on folate or folic acid supplementation could help establish causal relationships and confirm these suggestive associations. Such research would be especially relevant for populations at risk of folate deficiency and could enhance our understanding of folate’s role in immune function. Replicating this study in diverse populations with varying dietary habits, genetic backgrounds, and baseline health conditions would help verify the generalizability of these findings. Expanding research in these directions could significantly enhance our understanding of the effects of folate on the immune system and inform public health strategies for maintaining optimal immune function.

## Supplementary Information

Below is the link to the electronic supplementary material.

Supplementary Material 1.

## Data Availability

Data are not publicly available but can be applied for through an individual project agreement with the Chair of Epidemiology, Medical Faculty at University of Augsburg.

## References

[1] Iyer R, Tomar S (2009) Folate: a functional food constituent. J Food Sci 74(9):R114–R122 20492126 10.1111/j.1750-3841.2009.01359.x

[2] CDC. Folic acid: sources and recommended intake (2024). Available from: https://www.cdc.gov/folic-acid/about/intake-and-sources.html

[3] Cordero A et al (2010) CDC grand rounds: additional opportunities to prevent neural tube defects with folic acid fortification, MMWR: Morbidity & Mortality Weekly Report, 59(31) 20703205

[4] Eitenmiller RR, Landen W Jr, Ye L (2016) Vitamin analysis for the health and food sciences. CRC press

[5] Winkels RM et al (2007) Bioavailability of food folates is 80% of that of folic acid. Am J Clin Nutr 85(2):465–473 17284745 10.1093/ajcn/85.2.465

[6] Fertrin KY (2018) It is never too late to rethink serum folate. Hematol Transfus Cell Therapy 40(4):295–297 10.1016/j.htct.2018.07.002 PMC6200691 30370405

[7] Pfeiffer CM et al (2016) Applying inappropriate cutoffs leads to misinterpretation of folate status in the US population123. Am J Clin Nutr 104(6):1607–1615 27680995 10.3945/ajcn.116.138529 PMC5693380

[8] Mensink GBM, Weißenborn A, Richter A (2016) Folate Status in Germany. J Health Monit 1(2):24–28 10.17886/RKI-GBE-2016-040.2 PMC9838576 36654830

[9] Bailey LB, Gregory JF (1999) Folate metabolism and requirements12. J Nutr 129(4):779–782 10203550 10.1093/jn/129.4.779

[10] Rogers LM et al (2018) Global folate status in women of reproductive age: a systematic review with emphasis on methodological issues. Ann N Y Acad Sci 1431(1):35–57 30239016 10.1111/nyas.13963 PMC6282622

[11] Daly LE et al (1995) Folate levels and neural tube defects: implications for prevention. JAMA 274(21):1698–1702 7474275 10.1001/jama.1995.03530210052030

[12] Zhang X et al (2021) The association between folate and alzheimer’s disease: a systematic review and meta-analysis. Front NeuroSci 15:661198 33935641 10.3389/fnins.2021.661198 PMC8079632

[13] Pieroth R et al (2018) Folate and its impact on cancer risk. Curr Nutr Rep 7:70–84 30099693 10.1007/s13668-018-0237-y PMC6132377

[14] Liu JJ, Ward RL (2010) Folate and one-carbon metabolism and its impact on aberrant DNA methylation in cancer. Adv Genet 71:79–121 20933127 10.1016/B978-0-12-380864-6.00004-3

[15] Wagner C (2001) Biochemical role of folate in cellular metabolism. Clin Res Regul Affairs 18(3):161–180

[16] Depeint F et al (2006) Mitochondrial function and toxicity: role of B vitamins on the one-carbon transfer pathways. Chemico-Biol Interact 163(1–2):113–132 10.1016/j.cbi.2006.05.010 16814759

[17] Dhur A, Galan P, Hercberg S (1991) Folate status and the immune system. Prog Food Nutr Sci 15(1–2):43–60 1887065

[18] Klein L et al (2014) Positive and negative selection of the T cell repertoire: what thymocytes see (and don’t see). Nat Rev Immunol 14(6):377–391 24830344 10.1038/nri3667 PMC4757912

[19] Bonilla FA, Oettgen HC (2010) Adaptive immunity. J Allergy Clin Immunol 125(2):S33–S40 20061006 10.1016/j.jaci.2009.09.017

[20] Tian Y et al (2012) A novel splice variant of folate receptor 4 predominantly expressed in regulatory T cells. BMC Immunol 13:1–8 22694797 10.1186/1471-2172-13-30 PMC3724506

[21] Kunisawa J et al (2012) A pivotal role of vitamin B9 in the maintenance of regulatory T cells in vitro and in vivo. PLoS ONE 7(2):e32094 22363800 10.1371/journal.pone.0032094 PMC3282783

[22] Courtemanche CE-S, Mashiyama I, Kerry ST, Ames N, Bruce N (2004) Folate deficiency inhibits the proliferation of primary human CD8 + T lymphocytes in vitro. J Immunol 10.4049/jimmunol.173.5.3186 15322179

[23] Kinoshita M et al (2012) Dietary folic acid promotes survival of Foxp3 + regulatory T cells in the colon. J Immunol 189(6):2869–2878 22869901 10.4049/jimmunol.1200420

[24] Alnabbat KI et al (2022) High dietary folic acid intake is associated with genomic instability in peripheral lymphocytes of healthy adults. Nutrients 14(19):3944 36235597 10.3390/nu14193944 PMC9571807

[25] Hahn GJ (1977) The hazards of extrapolation in regression analysis. J Qual Technol 9(4):159–165

[26] Mahajan A et al (2019) Effect of imbalance in folate and vitamin B12 in maternal/parental diet on global methylation and regulatory MiRNAs. Sci Rep 9(1):17602 31772242 10.1038/s41598-019-54070-9 PMC6879517

[27] Scott JM (1999) Folate and vitamin B12. Proceedings of the nutrition society, 58(2): pp. 441–448 10.1017/s0029665199000580 10466189

[28] Baart A et al (2021) Relationship between intake and plasma concentrations of vitamin B12 and folate in 873 adults with a physically active lifestyle: a cross-sectional study. J Hum Nutr Dietetics 34(2):324–333 10.1111/jhn.12814 PMC8048838 32955764

[29] Semmler A et al (2010) Plasma folate levels are associated with the lipoprotein profile: a retrospective database analysis. Nutr J 9:1–4 20667074 10.1186/1475-2891-9-31 PMC2916887

[30] Wu C-H, Huang T-C, Lin B-F (2017) Folate deficiency affects dendritic cell function and subsequent T helper cell differentiation. J Nutr Biochem 41:65–72 28040582 10.1016/j.jnutbio.2016.11.008

[31] Berry RJ et al (2010) Fortification of flour with folic acid. FoodNutr Bull 31(1suppl1):S22–S35 10.1177/15648265100311S103 20629350

[32] Martiniak Y, Heuer T, Hoffmann I (2015) Intake of dietary folate and folic acid in Germany based on different scenarios for food fortification with folic acid. Eur J Nutr 54:1045–1054 25341394 10.1007/s00394-014-0781-1 PMC4575370

[33] Stover PJ (2004) Physiology of folate and vitamin B 12 in health and disease. Nutr Rev 62(suppl1):S3–S12 15298442 10.1111/j.1753-4887.2004.tb00070.x

[34] Duthie SJ, Hawdon A (1998) DNA instability (strand breakage, uracil misincorporation, and defective repair) is increased by folic acid depletion in human lymphocytes in vitro. FASEB J 12(14):1491–1497 9806758

