# Supplementary material for: Association between serum folate and T cell subsets in a population-based study
Source: Eur J Nutr. 2026 Jan 16;65(1):25. doi: 10.1007/s00394-025-03872-x (PMC12811324; doi:10.1007/s00394-025-03872-x)
Supplement: Supplementary file 1 — Supplementary Material 1. [file 394_2025_3872_MOESM1_ESM.docx]

# Supplements for Publication Manuscript

**Association between serum folate and T cell subsets in a population-based study**

First author: Laura Stoß

**Table of Contents**

**Supplementary Method** Flow Cytometry and Gating Strategy

**Tables**

Supplementary Table 1 Number of Knots Used in Linear and Log-linear Regression Models with both Sub-20 B9 and Threshold B9 covariates

Supplementary Table 2 Results of Linear, Log-linear, and Median regression analyses with Sub-20 B9 covariate

Supplementary Table 3 Regression Results of Linear, Log-linear, and Median regression analyses with Threshold B9 covariate

Supplementary Table 4 Sensitivity Analysis Results of Linear, Log-linear, and Median regression analyses with Sub-20 B9 covariate

Supplementary Table 5 Sensitivity Analysis Results of Linear, Log-linear, and Median regression analyses with Threshold B9 covariate

## Supplementary Method: Flow cytometry and gating strategy

Using venous EDTA whole blood samples, immunophenotyping of the participants was performed. Through flow cytometry, utilizing fluorescence-labeled antibodies (FACS, or fluorescence-activated cell sorting), different subpopulations of leukocytes were distinguished and quantified. The analyses were conducted on samples prepared with the DURAClone IM T Cell Subsets and DURAClone IM Treg kits from Beckman Coulter, using the Navios Flow Cytometer. Data analysis followed the manufacturer's instructions and was carried out using the Kaluza software, both provided by Beckman Coulter.

Only the percentage of gated cells from the DURAClone IM T Cell Subsets (tcs1) and DURAClone IM Treg (treg1) panels were analyzed in this study. The gating strategy for both panels are presented in *Figures 1* and *2*.

**
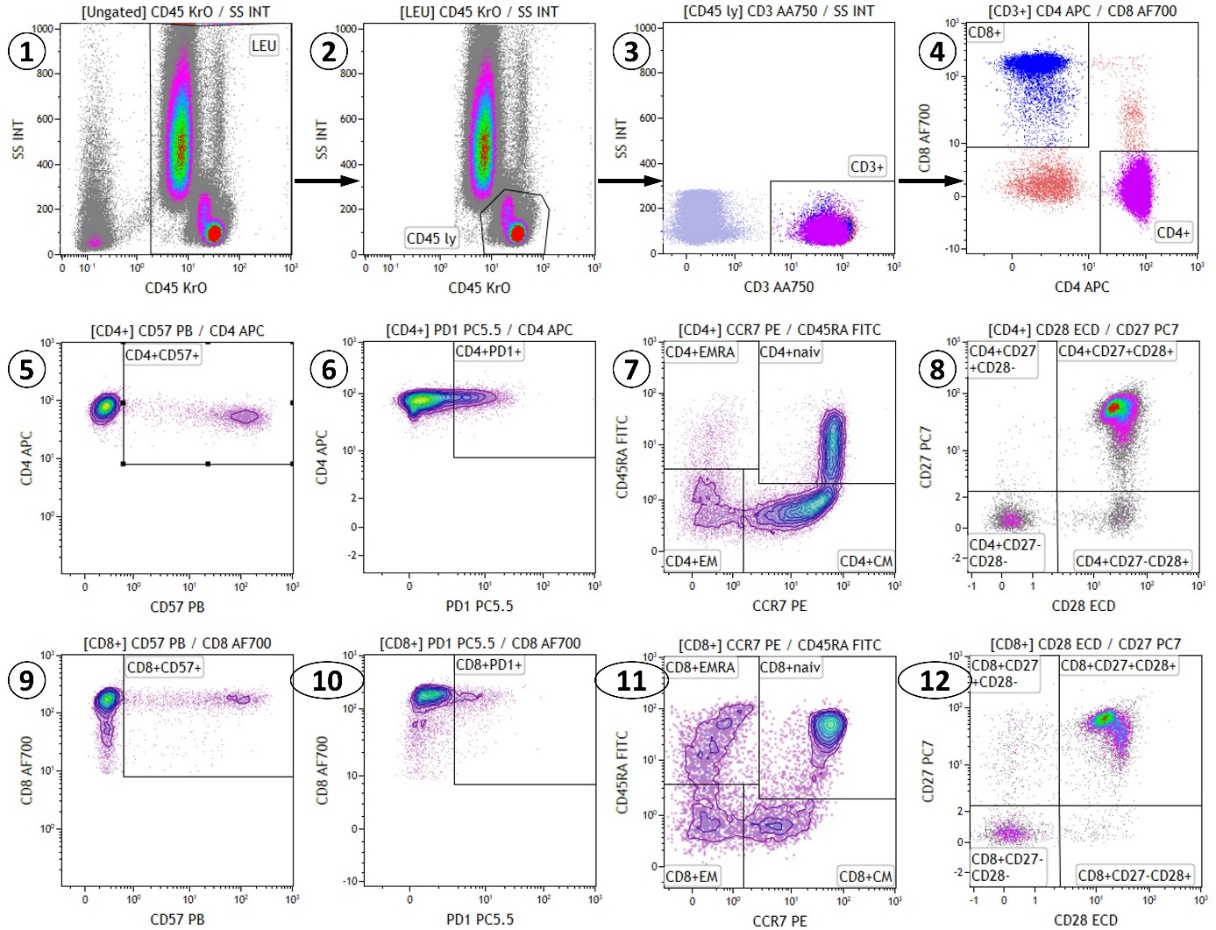
**

Figure 1: Gating Strategy for T Cell Subsets (Tcs Panel)

In the T Cell Subsets panel, leukocyte subpopulations were identified through a series of gating steps. Leukocytes were first separated from debris based on CD45 expression and side scatter intensity (SS INT) (1). Lymphocytes were then identified within the leukocyte population using the same markers (2). T lymphocytes were gated by CD3 expression (3), allowing further separation into CD4+ helper T cells and CD8+ cytotoxic T cells using CD4 and CD8 markers (4).

Within the CD4+ population, CD57+ T helper cells (senescent, terminally differentiated cells) (5) and PD1+ T helper cells (6) (indicative of immune exhaustion) were identified. T helper cells were further classified into naive, central memory, effector memory, and terminal effector cells based on CD45RA and CCR7 expression (7). CD27 and CD28 markers were used to assess T cell differentiation stages (8).

The same gating strategy was applied to CD8+ cytotoxic T cells (9, 10, 11, 12), providing a detailed characterization of T cell subsets. This approach allowed for the identification of functionally distinct T cell populations, crucial for understanding immune responses.

**
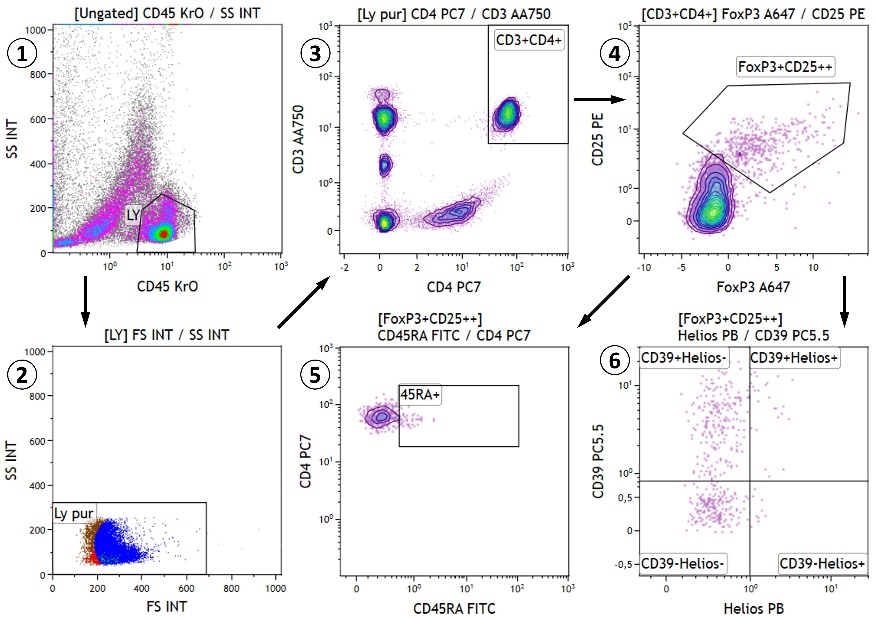
**

Figure 2: Gating Strategy for Regulatory T Cells (Treg Panel)

In this Treg flow cytometry analysis (Fig. 2), lymphocytes were identified based on CD45 expression, forward and side scatter intensity (1, 2). From this population, CD3+CD4+ T helper cells were isolated using CD3 and CD4 markers (3). Within the CD4+ T cells, CD25+ regulatory T cells (Tregs) were identified, and their expression of FoxP3, a specific marker for Tregs, was used to confirm their regulatory function (4). Natural regulatory T cells (nTregs) were identified by the expression of Helios, a transcription factor associated with thymus-derived Tregs, while CD39 expression was used to identify a subset of Tregs involved in immunosuppressive adenosine production (6). In addition, naive CD4+ T cells were characterized by CD45RA expression, indicating they had not yet been activated (5).

## Supplementary Tables

Supplementary Table 1: Number of Knots Used in Linear and Log-linear Regression Models with both Sub-20 B9 and Threshold B9 covariates

| **Outcome** | **Variable** | **# of knots** |
| --- | --- | --- |
| CD39+ Helios- Tregs | Age | 4 |
| CD4+ T cells | LDL cholesterol | 5 |
| CD4+ Central Memory T cells | Age | 4 |
| Naive CD4+ T cells | Age | 4 |
| CD8+ Central Memory T cells | Age | 3 |
| CD8+ Effector Memory T cells | Pack years | 3 |
| Naive CD8+ T cells | Age | 3 |
| CD8+ PD1+ T cells | LDL cholesterol | 3 |
| FOXP3+ CD25+ Tregs | Age | 3 |

Supplementary Table 2: Results of Linear, Log-linear, and Median regression analyses with Sub-20 B9 covariate

| **Outcome** | **Estimate** | **CI Lower** | **CI Upper** | **Regression Method** | ***p*-Value** | ***p*-Value adj.** |
| --- | --- | --- | --- | --- | --- | --- |
| CD8+ Effector Memory T cells | 0,394318286 | 0,086096 | 0,702541 | Linear | 0,012434269 | 0,186514035 |
| CD8+ CD57+ T cells | 0,192219839 | -0,16168 | 0,546115 | Linear | 0,287589856 | 0,677133462 |
| CD39+ Helios- Tregs | 0,100863895 | -0,29508 | 0,496809 | Linear | 0,617786037 | 0,826620726 |
| Naive CD4+ T cells | 0,090545884 | -0,22911 | 0,4102 | Linear | 0,578988726 | 0,826620726 |
| CD8+ T cells | 0,011548196 | -0,21765 | 0,240746 | Linear | 0,921371006 | 0,966202039 |
| FOXP3+ CD25+ Tregs | -0,020910582 | -0,06186 | 0,020043 | Linear | 0,317570232 | 0,68050764 |
| CD3+ CD4+ T cells | -0,030859076 | -0,20315 | 0,141434 | Linear | 0,725680466 | 0,887366107 |
| CD4+ T cells | -0,110131167 | -0,38014 | 0,159877 | Linear | 0,42438378 | 0,725298728 |
| CD8+ PD1+ T cells | -0,143492444 | -0,41059 | 0,123604 | Linear | 0,2934245 | 0,677133462 |
| CD4+ Central Memory T cells | -0,191387198 | -0,46854 | 0,085766 | Linear | 0,176460231 | 0,638068014 |
| CD8+ CD27+ CD28+ T cells | -0,268410141 | -0,67039 | 0,133565 | Linear | 0,191420404 | 0,638068014 |
| CD8+ Central Memory T cells | -0,287469163 | -0,47316 | -0,10178 | Linear | 0,00257218 | 0,077165397 |
| Naive CD8+ T cells | -0,330818619 | -0,6261 | -0,03554 | Linear | 0,028874474 | 0,216558556 |
| CD8+ CD27- CD28+ T cells | 1,017307893 | 1,002095 | 1,032752 | Log-linear | 0,026891889 | 0,216558556 |
| CD8+ Effector Memory RA+ T cells | 1,015029604 | 0,998394 | 1,031942 | Log-linear | 0,077824297 | 0,3335327 |
| CD8+ CD27- CD28- T cells | 1,013936258 | 0,989592 | 1,038879 | Log-linear | 0,26505954 | 0,677133462 |
| CD8+ CD27+ CD28- T cells | 1,012765444 | 1,000478 | 1,025204 | Log-linear | 0,042811623 | 0,25686974 |
| CD4+ CD27- CD28+ T cells | 1,004760127 | 0,992858 | 1,016805 | Log-linear | 0,435179237 | 0,725298728 |
| CD4+ Effector Memory T cells | 1,003047981 | 0,990582 | 1,015671 | Log-linear | 0,633742557 | 0,826620726 |
| CD4+ CD57+ T cells | 0,999746572 | 0,981373 | 1,018465 | Log-linear | 0,978643361 | 0,978643361 |
| CD4+ PD1+ T cells | 0,994706492 | 0,985891 | 1,003601 | Log-linear | 0,243585696 | 0,677133462 |
| Lymphocytes Tcs gate | 0,031220938 | -0,0417 | 0,104145 | Median | 0,401756667 | 0,725298728 |
| CD4+ Effector Memory RA+ T cells | 0,007253955 | -0,00805 | 0,022558 | Median | 0,353506519 | 0,707013037 |
| Lymphocytes Treg gate | 0,003559496 | -0,00652 | 0,013634 | Median | 0,488963042 | 0,772046908 |
| CD4+ CD27+ CD28- T cells | 0,000442379 | -0,00129 | 0,002171 | Median | 0,616219409 | 0,826620726 |
| CD4+ CD27- CD28- T cells | -0,001400686 | -0,03453 | 0,031734 | Median | 0,933995304 | 0,966202039 |
| CD39+ Helios+ Tregs | -0,006649172 | -0,08233 | 0,069036 | Median | 0,863463121 | 0,959403467 |
| Leucocytes | -0,020900697 | -0,24574 | 0,203935 | Median | 0,855508893 | 0,959403467 |
| CD4+ CD27+ CD28+ T cells | -0,028071356 | -0,19329 | 0,137148 | Median | 0,739471756 | 0,887366107 |
| CD39- Helios+ Tregs | -0,057093001 | -0,11649 | 0,002301 | Median | 0,060116544 | 0,300582721 |

Regression models were adjusted for age, sex, education (International Standard Classification of Education ISCED3C), smoking intensity (pack years: number of packs smoked per day multiplied with the number of years), alcohol consumption (AUDIT score), relative body fat content, and Low-Density Lipoprotein (LDL) cholesterol levels.

Supplementary Table 3: Regression Results of Linear, Log-linear, and Median regression analyses with Threshold B9 covariate

| **Outcome** | **Estimate** | **CI Lower** | **CI Upper** | **Regression Method** | ***p*-Value** | ***p*-Value adj.** |
| --- | --- | --- | --- | --- | --- | --- |
| CD8+ CD27+ CD28+ T cells | 4,865298 | -0,95024 | 10,68084 | Linear | 0,101797 | 0,856557518 |
| CD39+ Helios- Tregs | 3,650396 | -2,05164 | 9,352433 | Linear | 0,210057 | 0,856557518 |
| CD4+ T cells | 2,924139 | -1,06999 | 6,918273 | Linear | 0,152062 | 0,856557518 |
| CD8+ Central Memory T cells | 1,604561 | -1,05354 | 4,262667 | Linear | 0,23727 | 0,856557518 |
| CD3+ CD4+ T cells | 1,473419 | -1,08715 | 4,033991 | Linear | 0,259872 | 0,856557518 |
| CD8+ Effector Memory T cells | 1,069696 | -3,414 | 5,553396 | Linear | 0,640239 | 0,979332226 |
| CD8+ PD1+ T cells | 0,896111 | -2,83961 | 4,631836 | Linear | 0,638445 | 0,979332226 |
| Naive CD8+ T cells | 0,8253 | -3,35297 | 5,003571 | Linear | 0,698797 | 0,979332226 |
| Naive CD4+ T cells | 0,720691 | -3,8813 | 5,322685 | Linear | 0,758996 | 0,979332226 |
| FOXP3+ CD25+ Tregs | 0,056584 | -0,52412 | 0,637291 | Linear | 0,848606 | 0,979332226 |
| CD4+ Central Memory T cells | -0,30854 | -4,30776 | 3,690676 | Linear | 0,879858 | 0,979332226 |
| CD8+ T cells | -1,77979 | -5,16131 | 1,601732 | Linear | 0,303076 | 0,856557518 |
| CD8+ CD57+ T cells | -3,09385 | -8,25309 | 2,065395 | Linear | 0,240437 | 0,856557518 |
| CD8+ CD27- CD28+ T cells | 1,011351 | 0,822081 | 1,244199 | Log-linear | 0,915007 | 0,979332226 |
| CD4+ CD27- CD28+ T cells | 0,990674 | 0,833511 | 1,17747 | Log-linear | 0,91537 | 0,979332226 |
| CD4+ PD1+ T cells | 0,936938 | 0,825433 | 1,063507 | Log-linear | 0,314071 | 0,856557518 |
| CD4+ Effector Memory T cells | 0,930196 | 0,779348 | 1,110241 | Log-linear | 0,423151 | 0,976502732 |
| CD4+ CD57+ T cells | 0,924414 | 0,708173 | 1,206685 | Log-linear | 0,56342 | 0,979332226 |
| CD8+ CD27+ CD28- T cells | 0,873125 | 0,737641 | 1,033495 | Log-linear | 0,115333 | 0,856557518 |
| CD8+ Effector Memory RA+ T cells | 0,832792 | 0,659203 | 1,052092 | Log-linear | 0,125552 | 0,856557518 |
| CD8+ CD27- CD28- T cells | 0,748828 | 0,530442 | 1,057125 | Log-linear | 0,100691 | 0,856557518 |
| Leucocytes | 1,09649 | -1,57094 | 3,763925 | Median | 0,42075 | 0,976502732 |
| CD4+ CD27+ CD28+ T cells | 0,377812 | -1,67544 | 2,431062 | Median | 0,71849 | 0,979332226 |
| CD39+ Helios+ Tregs | 0,3127 | -0,76756 | 1,392958 | Median | 0,570689 | 0,979332226 |
| CD4+ Effector Memory RA+ T cells | 0,027629 | -0,19751 | 0,252771 | Median | 0,810004 | 0,979332226 |
| CD4+ CD27- CD28- T cells | 0,015949 | -0,45135 | 0,483245 | Median | 0,946688 | 0,979332226 |
| Lymphocytes Tcs gate | 0,009336 | -1,07844 | 1,097115 | Median | 0,986585 | 0,986584575 |
| CD4+ CD27+ CD28- T cells | -0,00092 | -0,02526 | 0,023423 | Median | 0,940913 | 0,979332226 |
| Lymphocytes Treg gate | -0,0214 | -0,16535 | 0,122539 | Median | 0,770816 | 0,979332226 |
| CD39- Helios+ Tregs | -0,05808 | -0,92339 | 0,807219 | Median | 0,895373 | 0,979332226 |

Regression models were adjusted for age, sex, education (International Standard Classification of Education ISCED3C), smoking intensity (pack years: number of packs smoked per day multiplied with the number of years), alcohol consumption (AUDIT score), relative body fat content, and Low-Density Lipoprotein (LDL) cholesterol levels.

Supplementary Table 4: Sensitivity Analysis Results of Linear, Log-linear, and Median regression analyses with Sub-20 B9 covariate

| **Outcome** | **Estimate** | **CI Lower** | **CI Upper** | **Regression Method** | ***p*-Value** | ***p*-Value adj.** |
| --- | --- | --- | --- | --- | --- | --- |
| CD8+ Effector Memory T cells | 0,368097993 | 0,053375 | 0,682821 | Linear | 0,022244 | 0,182141561 |
| CD8+ CD57+ T cells | 0,192219839 | -0,16168 | 0,546115 | Linear | 0,28759 | 0,762312878 |
| CD39+ Helios- Tregs | 0,116103126 | -0,28354 | 0,515747 | Linear | 0,569324 | 0,80220623 |
| Naive CD4+ T cells | 0,108060661 | -0,2219 | 0,438025 | Linear | 0,521223 | 0,80220623 |
| CD8+ T cells | 0,063047298 | -0,18253 | 0,308623 | Linear | 0,615025 | 0,80220623 |
| CD3+ CD4+ T cells | -0,01722222 | -0,20284 | 0,168399 | Linear | 0,855824 | 0,893491353 |
| FOXP3+ CD25+ Tregs | -0,032669866 | -0,08091 | 0,015574 | Linear | 0,185078 | 0,680084051 |
| CD4+ Central Memory T cells | -0,119915905 | -0,41914 | 0,179313 | Linear | 0,432512 | 0,763255709 |
| CD4+ T cells | -0,129578692 | -0,40686 | 0,147704 | Linear | 0,360097 | 0,763255709 |
| CD8+ PD1+ T cells | -0,138371229 | -0,42366 | 0,146917 | Linear | 0,34262 | 0,763255709 |
| CD8+ CD27+ CD28+ T cells | -0,263570792 | -0,66958 | 0,142437 | Linear | 0,204025 | 0,680084051 |
| CD8+ Central Memory T cells | -0,285052075 | -0,47873 | -0,09138 | Linear | 0,004109 | 0,123259719 |
| Naive CD8+ T cells | -0,345034423 | -0,64383 | -0,04624 | Linear | 0,024286 | 0,182141561 |
| CD8+ CD27- CD28+ T cells | 1,01923269 | 1,003735 | 1,03497 | Log-linear | 0,015617 | 0,182141561 |
| CD8+ Effector Memory RA+ T cells | 1,015029604 | 0,998394 | 1,031942 | Log-linear | 0,077824 | 0,389121483 |
| CD8+ CD27- CD28- T cells | 1,012874642 | 0,988455 | 1,037897 | Log-linear | 0,304925 | 0,762312878 |
| CD8+ CD27+ CD28- T cells | 1,012235861 | 0,999366 | 1,025271 | Log-linear | 0,063747 | 0,382480695 |
| CD4+ CD27- CD28+ T cells | 1,003527728 | 0,991169 | 1,016041 | Log-linear | 0,577763 | 0,80220623 |
| CD4+ Effector Memory T cells | 1,002746211 | 0,989961 | 1,015697 | Log-linear | 0,675571 | 0,844464219 |
| CD4+ CD57+ T cells | 0,998036627 | 0,978916 | 1,017531 | Log-linear | 0,84223 | 0,893491353 |
| CD4+ PD1+ T cells | 0,994746949 | 0,985606 | 1,003973 | Log-linear | 0,264656 | 0,762312878 |
| Lymphocytes Tcs gate | 0,033921583 | -0,04596 | 0,113799 | Median | 0,405633 | 0,763255709 |
| CD4+ Effector Memory RA+ T cells | 0,00644173 | -0,00963 | 0,022514 | Median | 0,432451 | 0,763255709 |
| Lymphocytes Treg gate | 0,003862116 | -0,00732 | 0,015045 | Median | 0,498729 | 0,80220623 |
| CD4+ CD27+ CD28- T cells | 0,000304602 | -0,00148 | 0,002092 | Median | 0,738484 | 0,852097213 |
| CD4+ CD27- CD28- T cells | -0,000580868 | -0,03764 | 0,036479 | Median | 0,975503 | 0,97550302 |
| CD39+ Helios+ Tregs | -0,020038491 | -0,09764 | 0,057565 | Median | 0,613166 | 0,80220623 |
| Leucocytes | -0,023909784 | -0,29604 | 0,248224 | Median | 0,863708 | 0,893491353 |
| CD4+ CD27+ CD28+ T cells | -0,030167115 | -0,19039 | 0,13006 | Median | 0,71225 | 0,852097213 |
| CD39- Helios+ Tregs | -0,054379335 | -0,1202 | 0,011444 | Median | 0,107414 | 0,460346083 |

Regression models were adjusted for age, sex, education (International Standard Classification of Education ISCED3C), smoking intensity (pack years: number of packs smoked per day multiplied with the number of years), alcohol consumption (AUDIT score), relative body fat content, and Low-Density Lipoprotein (LDL) cholesterol levels. Only extreme values categorized as outliers were removed by the principal investigator.

Supplementary Table 5: Sensitivity Analysis Results of Linear, Log-linear, and Median regression analyses with Threshold B9 covariate

| **Outcome** | **Estimate** | **CI Lower** | **CI Upper** | **Regression Method** | ***p*-Value** | ***p*-Value adj.** |
| --- | --- | --- | --- | --- | --- | --- |
| CD8+ CD27+ CD28+ T cells | 5,028486 | -0,84207 | 10,89905 | Linear | 0,093911 | 0,901638495 |
| CD39+ Helios- Tregs | 3,568412 | -2,1911 | 9,327919 | Linear | 0,225098 | 0,901638495 |
| CD4+ T cells | 2,938699 | -1,17473 | 7,052127 | Linear | 0,162181 | 0,901638495 |
| CD8+ Central Memory T cells | 1,302136 | -1,50713 | 4,111397 | Linear | 0,364022 | 0,966529264 |
| CD8+ Effector Memory T cells | 0,744312 | -3,8298 | 5,31842 | Linear | 0,749887 | 0,966529264 |
| Naive CD8+ T cells | 0,671641 | -3,57778 | 4,921062 | Linear | 0,756838 | 0,966529264 |
| Naive CD4+ T cells | 0,430293 | -4,33898 | 5,199562 | Linear | 0,859699 | 0,966529264 |
| CD8+ PD1+ T cells | 0,326343 | -3,70271 | 4,355397 | Linear | 0,873923 | 0,966529264 |
| CD4+ Central Memory T cells | 0,211535 | -4,11594 | 4,53901 | Linear | 0,923705 | 0,966529264 |
| CD3+ CD4+ T cells | 0,105527 | -2,51198 | 2,723034 | Linear | 0,937045 | 0,966529264 |
| FOXP3+ CD25+ Tregs | -0,09953 | -0,79202 | 0,592957 | Linear | 0,778265 | 0,966529264 |
| CD8+ T cells | -2,31145 | -5,97875 | 1,355853 | Linear | 0,217543 | 0,901638495 |
| CD8+ CD57+ T cells | -3,09385 | -8,25309 | 2,065395 | Linear | 0,240437 | 0,901638495 |
| CD8+ CD27- CD28+ T cells | 1,024152 | 0,827425 | 1,267652 | Log-linear | 0,826502 | 0,966529264 |
| CD4+ CD27- CD28+ T cells | 0,99614 | 0,831585 | 1,193258 | Log-linear | 0,966529 | 0,966529264 |
| CD4+ PD1+ T cells | 0,982258 | 0,864376 | 1,116216 | Log-linear | 0,783838 | 0,966529264 |
| CD4+ Effector Memory T cells | 0,954306 | 0,795553 | 1,144737 | Log-linear | 0,61457 | 0,966529264 |
| CD4+ CD57+ T cells | 0,870159 | 0,656854 | 1,152731 | Log-linear | 0,332765 | 0,966529264 |
| CD8+ CD27+ CD28- T cells | 0,852521 | 0,713723 | 1,01831 | Log-linear | 0,078956 | 0,901638495 |
| CD8+ Effector Memory RA+ T cells | 0,832792 | 0,659203 | 1,052092 | Log-linear | 0,125552 | 0,901638495 |
| CD8+ CD27- CD28- T cells | 0,738165 | 0,522082 | 1,043684 | Log-linear | 0,086343 | 0,901638495 |
| Leucocytes | 1,48504 | -1,4739 | 4,443983 | Median | 0,325673 | 0,966529264 |
| CD4+ CD27+ CD28+ T cells | 0,313356 | -1,82348 | 2,450198 | Median | 0,773895 | 0,966529264 |
| CD39+ Helios+ Tregs | 0,191238 | -0,95293 | 1,335401 | Median | 0,743336 | 0,966529264 |
| CD4+ CD27- CD28- T cells | 0,018814 | -0,49526 | 0,532886 | Median | 0,942838 | 0,966529264 |
| CD4+ CD27+ CD28- T cells | -0,00177 | -0,02696 | 0,023429 | Median | 0,890831 | 0,966529264 |
| CD4+ Effector Memory RA+ T cells | -0,00639 | -0,2447 | 0,231917 | Median | 0,958102 | 0,966529264 |
| Lymphocytes Treg gate | -0,01822 | -0,17778 | 0,141345 | Median | 0,822994 | 0,966529264 |
| Lymphocytes Tcs gate | -0,04992 | -1,22347 | 1,123627 | Median | 0,933578 | 0,966529264 |
| CD39- Helios+ Tregs | -0,05544 | -0,96018 | 0,849308 | Median | 0,904449 | 0,966529264 |

Regression models were adjusted for age, sex, education (International Standard Classification of Education ISCED3C), smoking intensity (pack years: number of packs smoked per day multiplied with the number of years), alcohol consumption (AUDIT score), relative body fat content, and Low-Density Lipoprotein (LDL) cholesterol levels. Only extreme values categorized as outliers were removed by the principal investigator.
